# Adolescents’ impairment due to climate anxiety is associated with self-efficacy and behavioral engagement: a cross-sectional analysis in Quebec (Canada)

**DOI:** 10.1186/s12889-024-20333-y

**Published:** 2024-10-30

**Authors:** Anne-Marie Turcotte-Tremblay, Gabrielle Fortier, Richard E. Bélanger, Claude Bacque Dion, Rabi Joel Gansaonré, Scott T. Leatherdale, Slim Haddad

**Affiliations:** 1 COMPASS-Québec, Centre de Recherche VITAM, CIUSSS de la Capitale-Nationale, 2480 Chemin de la Canardière, Quebec City, QC G1J 2G1 Canada; 2 Faculté des sciences infirmières, Université Laval, Quebec City, QC Canada; 3 Faculté de médecine, Université Laval, Québec City, QC Canada; 4 School of Public Health Sciences, University of Waterloo, Waterloo, ON Canada; 5 Direction de santé publique du CIUSSS de la Capitale-Nationale, Quebec City, QC Canada

**Keywords:** Climate anxiety, Self-efficacy, Climate change, Adolescents

## Abstract

**Background:**

The current understanding of climate anxiety among adolescents is sparse. This study identifies the characteristics of adolescents living in Quebec (Canada) who experience impairments induced by climate anxiety, who have feelings of self-efficacy and who adopt pro-environment behaviors. It also characterizes the association between climate anxiety, feelings of self-efficacy, and environmental actions.

**Methods:**

We used a cross-sectional design to analyze data from the COMPASS study on adolescent health. Participants (*n* = 45 362) from 113 schools in Quebec (Canada) answered questions about climate anxiety, self-efficacy, behavioral engagement, and several indicators of well-being. Data were analyzed using ordered logistic regressions adjusted for potential confounders (age, gender, school type, material deprivation, etc.).

**Results:**

9% of adolescents reported that thinking about climate change made it difficult to sleep at least sometimes. 6% of adolescents reported that thinking about climate change interfered with their ability to get work or assignments done at least sometimes. Only 34% believed they could do something to address the problem, and 43% tried to reduce their behaviors that contribute to climate change at least sometimes. Adolescents who were boys or who were less anxious experienced less impairment and were less involved in the fight against climate change. Adolescents from less affluent families experienced more difficulty sleeping and interference with work but were not more engaged. Those with stronger school connectedness experienced less impairment and were more often involved in the fight against climate change. Adolescents who felt they could do something about climate change were more likely to try to reduce behaviors that contribute to climate change.

**Conclusion:**

The findings are useful to identify and support vulnerable groups that are more likely to experience impairment due to climate anxiety. The feeling of climate self-efficacy was not well developed among some groups of adolescents. Improving adolescents’ beliefs in their capacity to help address climate change may be a key strategy to promote pro-environmental actions. As the threat of climate change increases, it will be important to follow the evolution of climate anxiety and engagement among adolescents.

**Supplementary Information:**

The online version contains supplementary material available at 10.1186/s12889-024-20333-y.

## Introduction

Research suggests that the threats of climate change, increasing pressure to act, and collective inaction can lead to anxiety and distress, even among people who have not personally experienced the direct impacts of climate change [1]. Terms used to describe this phenomenon include climate anxiety, eco anxiety, and eco distress [1, 2]. Climate anxiety has been defined as the wide range of negative responses associated with concerns about climate change [3]. It is not considered to be a diagnosis or mental illness but can affect mental health over time [4]. While this state can stimulate adaptive responses (for example, reducing one’s carbon footprint), it has the potential to cause impairment [1].

According to Clayton and Karazsia, climate anxiety comprises two factors: cognitive-emotional impairment and functional impairment [5]. Cognitive-emotional impairment can include difficulty concentrating, difficulty sleeping, having nightmares about climate change, or crying because of climate change [5]. Functional impairment can include having concerns about climate change that make it hard to have fun with family or friends, or concerns that interfere with one’s ability to get work or school assignments done [5]. Clayton and Karazsia also explain that the emotional response to climate change can be related to behavioral engagement (as a consequence) [5].

Past studies have shown climate anxiety is higher among certain groups [6]. For example, studies on adults found climate change anxiety to be associated with poorer well-being, general anxiety, depression, and coronavirus anxiety [6–10]. Gago et al.’s [11] meta-analysis showed that climate anxiety has a significant negative correlation with psychological wellbeing (i.e., anxiety, depression, trauma symptoms, adjustment disorders) but there is still a large portion of variance that is not explained. Some studies have reported that younger adults and lonely ones have higher climate anxiety scores than do others [5, 6, 10, 12]. Gender does not appear to predict climate anxiety, but women tend to be more engaged [5, 6, 13].

There is a consensus in the literature that the current understanding of climate anxiety in children and adolescents is sparse, but some empirical evidence is beginning to emerge [1, 14]. In a study by Hickman et al. [14] of young people (aged 16–25 years) in 10 countries (Australia, Brazil, Finland, France, India, Nigeria, Philippines, Portugal, the United Kingdom, and the United States), 59% of respondents were very or extremely worried and 84% were at least moderately worried. Climate anxiety and distress were correlated with perceived inadequate government response and associated with feelings of betrayal [14]. Some research suggests that young people with pre-existing mental illness and lacking social support may be at elevated risk for climate change-related mental health effects [15]. School connectedness, school type and family affluence have also been shown to influence a wide range of behaviors and health outcomes amongst adolescents, although their relation to climate anxiety and engagement have not been sufficiently explored [16–20]. A systematic review found that school connectedness is a protective factor for general anxiety symptoms, but it is not clear if it also protects against climate-related anxiety [21]. Based on their literature review, Crandon et al. suggest that future research on climate anxiety in adolescents should explore psychological traits, and youth engagement in policy [1]. Adopting a social-ecological perspective, these authors highlight the need to consider factors at various levels: individual (e.g., age, gender, anxiety, school connectedness), family (e.g., family affluence), and school (e.g., public vs. private, rural vs. urban) [1].

Evidence also suggests there are regional and contextual differences in climate risk perceptions [22]. Most regions of the world are underrepresented in the literature, with recent findings coming primarily from Australia, Finland, and the US [23]. Thus, it is unclear whether the results of previous studies are applicable to Canada, a country previously ranked among the top 10 countries most affected by climate change [24]. Experts argue there is a need for research among a greater diversity of people and places [2, 23].

According to Clayton [25], climate anxiety could be a source of motivation to encourage behavioral engagement with the issue of climate change. Alternatively, climate anxiety could be a source of eco-paralysis that inhibits people from taking effective action [25, 26]. Studies on adults found that climate anxiety was positively linked to some types of pro-environmental action, such as the adoption of green and sustainable behaviors [6, 7, 13]. Similarly, a study on 150 adolescents in Switzerland found that being worried about climate change has the potential to translate into climate action [27].

An individual’s assessments of their ability to cope with climate change (e.g., climate self-efficacy) may be related to climate anxiety or pro-environmental action, or both [25]. In their review, Li et al. [28] highlight that perceived behavioral control may influence pro-environmental behaviours (e.g., the intention to recycle batteries). A study conducted using data collected from a sample of 259 Dutch adolescents revealed a significant positive relationship between environmental self-efficacy and pro-environmental behavior [29]. In the United States, a study on 848 high school students living in the West Coast found that adolescents who have higher levels of perceived self-efficacy reported more pro-environmental behaviors than adolescents who have lower levels of perceived self-efficacy [30].

Climate self-efficacy and pro-environmental behavior have also been associated with certain demographic and psychological variables [31]. In their narrative review, Li et al. [28] suggest that women, highly educated young people with good income levels, married couples, and urban residents tend to exhibit more environmental behavioral intentions. To date, however, the evidence on climate self-efficacy and pro-environmental behavior amongst adolescents remains scarce. It is not clear whether adolescents in Quebec (Canada) currently believe they can do something to address climate change and if so, whether they can leverage that belief into environmental actions [32].

This paper is intended to fill a knowledge gap on climate anxiety, climate self-efficacy and pro-environmental behavior amongst youth. More specifically this study aimed to identify the main factors related to the following conditions among adolescents in Quebec (Canada): (1) feeling impaired due to climate anxiety ; (2) believing in their ability to contribute to the fight against climate change ; and (3) being committed to fighting climate change. The research questions were: (1) Do a set of demographic variables (e.g., gender, family affluence) predict climate anxiety, self-efficacy, and pro-environmental behavior? (2) Do a set of markers of well-being (e.g., anxiety symptoms, ability to talk to family and school connectedness) predict climate anxiety, self-efficacy, and pro-environmental behavior, and (3) How are climate anxiety, self-efficacy, and pro-environmental behavior related to each other?

Based on the literature described above, we hypothesized that adolescents who have the worst indicators of well-being and mental health [6–10], and those who are less connected to their school or who talk less to their families about their problems are more likely to have climate-related impairments compared to others [5, 6, 10, 12, 15]. We also hypothesized that girls would be more likely to change their behaviors compared to others [5, 6, 13]. Next, we hypothesized that those who have impairment due to climate anxiety are more likely to believe in their ability to help solve the problem and to change their behaviors [6, 7, 13]. Lastly, we hypothesized that adolescents who have a sense of climate self-efficacy are more likely to engage in environmental actions at least sometimes [25]. Given the current state of the literature and theorizing, we did not advance additional hypotheses.

## Methods

### Survey design and study population

This study was based on a cross-sectional design. Data were collected as part of the COMPASS longitudinal study (https://uwaterloo.ca/compass-system/). COMPASS is a multicentre study of adolescent health in Canada. Each year, youth in participating high schools are asked to complete a questionnaire about their lifestyle and behaviors. In the province of Quebec, school surveys have been conducted since the spring of 2017 in partnership with school communities and the regional public health departments. Since 2020, data collection has been conducted online using Qualtrics XM (Seattle, WA, USA).

The current analysis is based on data collected between March and May 2022. The study population included all students in the five years of the high school level (equivalent to Grades 7 to 11 in the US and the rest of Canada) of 113 secondary schools in the eastern region of the province of Quebec (Canada). Of the 58,792 adolescents solicited, 48 289 (82.1%) answered the online questionnaire. Parents’ active refusal rate was less than 0.56% (327 participants).

### Measures

Measures related to climate change anxiety were self-reported and taken from Clayton and Karazsia’s questionnaire [5]. In French-language schools, a validated translation of the questionnaire was used [33]. Two questions addressed youth concerns. The first question assessed cognitive-emotional impairment related to climate change [33]. Using a 5-point scale, participants reported how often thinking about climate change made it difficult for them to sleep. The second question was a measure of functional impairment [33]. Using the same 5-point scale, participants reported the extent to which their concerns about climate change undermined their ability to work to their potential. Two other questions dealt with behavioral engagement [33]. One asked how often they tried to reduce their behaviors that contributed to climate change; the other asked how often they believed they could do something to help address the problem of climate change. The latter served as a measure of psychosocial self-efficacy, which refers to one’s perceived ability to engage in various situation-specific self-management tasks [34]. For these four variables, the response options were: “never”, “rarely”, “sometimes”, “often” or “almost always”. The response options “often” and “almost always” were combined due to the small sample size in these categories and the similarity of patterns observed.

The adolescents’ characteristics included age and gender (male, female vs. neither male nor female). We used a score of family affluence based on adolescents’ responses regarding: (1) the average amount of money they received each week for personal spendings or savings; (2) skipping breakfast because there was nothing to eat at home; (3) going to bed hungry at night because there was not enough money to buy food; (4) having the feeling that they and their family were less financially comfortable than the average student in their class; (5) having their own bedroom; (6) the number of people in their household; and (7) being worried about their family’s ability to pay bills and expenses. Composite scores were dichotomized into two groups (more affluent = 0 vs. less affluent = 1). Adolescents in the last two quintiles were considered to be less affluent.

Measures of well-being were: (1) anxiety; (2) school connectedness; and (3) being able to talk about their problems with family.

Anxiety symptoms were measured using the 7-item Generalized Anxiety Disorder scale [35]. Students were asked how often they had experienced each symptom in the past two weeks. To account for non-linearity, a quadratic term for anxiety symptoms was also included.

School connectedness was collected using a modified version of the National Longitudinal Study of Adolescent Health School Connectedness scale [36, 37]. Items in this scale attempt to tap into overall school connectedness by capturing the adolescent’s feelings of belonging, liking/enjoyment, closeness, fair treatment, and safety [38]. To account for non-linearity, a quadratic term for school connectedness was also included.

Participants used a 5-point Likert scale to rate the extent to which they could talk about their problems with their family (strongly agree, agree, neither agree or disagree, disagree, strongly disagree).

School characteristics included the type of school attended (public = 0 vs. private = 1) and the location (rural = 0 vs. urban = 1).

### Statistical analyses

Descriptive statistics were calculated. The four outcomes were: (1) concerns about climate change that interfered with sleep, (2) concerns about climate change that interfered with ability to work; (3) the belief that they could do something to help address the problem of climate change; and 3) trying to reduce behaviors that contribute to climate change.

We performed ordered logistic regressions to examine associations between the ordinal outcomes and participants’ characteristics as well as indicators of well-being. We also examined associations between climate anxiety (interference with sleep or ability to work), self-efficacy (the belief that they could do something to address the problem), and behavioral engagement (trying to reduce climate-negative behaviors). Potential confounders included in multiple regressions were age, gender, school type, family affluence, and location.

All models were performed using STATA software version 17 [39]. Multiple imputation was used in order to handle missing data. Stata’s software MI command was used to fit the ordered logistic regression model on the imputation datasets [40, 41]. Robust error variance estimators were used to control for possible overdispersion (variance in the response greater than what is assumed by the model) and clustering of observations. The predicted values were estimated using Stata.

## Results

### Descriptive statistics

The study sample size was 45 362 respondents across 113 schools in Quebec (Canada). The flow chart describing the sample size for each outcome variable is available in Figure S1 (see Supplementary file 1). Table 1 presents descriptive statistics of the adolescents. About half of the adolescents were female. Likewise, about half of the respondents were 14 years of age or less. Public schools made up 85% of the sample, and urban schools, 71%. The mean score for anxiety symptoms was 6.8%. Also, 60% of the adolescents either strongly agreed or agreed that they could talk to their family about their problems. The mean score for school connectedness was 18.3.

9% of respondents reported that thinking about climate change made it difficult to sleep at least sometimes. 6% of respondents reported that thinking about climate change interfered with their ability to get work or assignments done at least sometimes. Just over one-third (34%) believed they could do something to address the problem of climate change either sometimes, often, or almost always. Approximately 43% of adolescents tried to change their behaviors that contributed to climate change either sometimes, often, or almost always.

**Table 1:** Descriptive statistics of adolescents in the COMPASS Quebec study in 2022

Variables	*N* (%)
**Age (years)**	
≤ 12 or younger	4 844 (11%)
13	10 495 (23%)
14	9 773 (22%)
15	8 593 (19%)
16	7 518 (17%)
≥ 17	4 073 (9%)
**Gender**	
Male	21 237 (47%)
Female	21 798 (48%)
Other than male or female	2 221 (5%)
**Family affluence**	
More affluent	27 472 (61%)
Less affluent	17 849 (39%)
**School location**	
Rural	12 929 (29%)
Urban	32 433 (71%)
**School type**	
Public	38 741 (85%)
Private	6 621 (15%)
**School connectedness (0–24), mean (SD)**	18.3 (3.4)
**Anxiety level (0–21), mean (SD)**	6.8 (5.6)
**Can talk to family about problems**	
Strongly agree	14 500 (32%)
Agree	12 685 (28%)
Neither agree nor disagree	9 167 (20%)
Disagree	5 073 (11%)
Strongly disagree	3 585 (8%)
**Difficulty sleeping**	
Never	3 5018 (77%)
Rarely	6 274 (14%)
Sometimes	2 669 (6%)
Often or almost always	1 315 (3%)
**Interference with ability to work**	
Never	37 482 (83%)
Rarely	4 940 (11%)
Sometimes	1 746 (4%)
Often or almost always	1 019 (2%)
**Believe they can do something**	
Never	20 328 (45%)
Rarely	9 254 (21%)
Sometimes	8 877 (20%)
Often or almost always	6 629(14%)
**Try to change behaviors**	
Never	19 441 (43%)
Rarely	6 228 (14%)
Sometimes	10 387 (23%)
Often or almost always	9 089 (20%)

### Environmental concerns interfering with adolescents’ lives

Table 2 shows the adjusted proportions of climate anxiety in accordance with the adolescents’ characteristics. In comparison to boys, girls and those who did not identify as male or female were more likely to experience difficulty sleeping (respectively, RR = 1.30; CI95 [1.22, 1.38] and RR = 2.13; CI95 [1.94, 2.43]). Girls and those who did not identify as male or female were also more likely than boys to experience interference with work or schoolwork (respectively, RR = 1.14; CI95 [1.07, 1.21] and RR = 2.12; CI95 [1.89, 2.35]). It is noteworthy that those who did not identify as male or female had more than two times the risk of experiencing cognitive-emotional impairment and functional impairment induced by climate anxiety, compared to boys.

Adolescents from less affluent families experienced more difficulty sleeping and interference with work or schoolwork (respectively, RR = 1.32; CI95 [1.26, 1.38] and RR = 1.34.; CI95 [1.27, 1.42]). These considerable effect sizes suggest that adolescents who were less affluent were more than 30% more likely to experience impairment due to climate anxiety. Being able to talk to their family about their problems was not meaningfully associated with difficulty sleeping or interference with work (respectively, RR = 0.91; CI95 [0.82, 1.00] and RR = 0.95; CI95 [0.86, 1.04]).

**Table 2:** Predicted values and risk ratios for climate anxiety amongst adolescents in Quebec (Canada)

	Difficulty sleeping								Interference with ability to work								
	Never				Often/almost always					Never				Often/almost always			
	Predicted proportion		95% CI		Predicted proportion		95% CI			Predicted proportion		95% CI		Predicted proportion		95% CI	
**Gender**																	
Boys (ref)	0.80		0.79	0.81	0.02		0.02	0.03		0.84		0.84	0.85	0.02		0.02	0.02
Girls	0.76		0.75	0.77	0.03		0.03	0.03		0.83		0.82	0.84	0.02		0.02	0.02
aRR	0.95		0.93	0.96	1.30		1.22	1.38		0.98		0.97	0.99	1.14		1.07	1.21
Neither boy nor girl*	0.65		0.63	0.68	0.05		0.05	0.06		0.72		0.70	0.74	0.04		0.04	0.05
aRR	0.82		0.79	0.84	2.13		1.94	2.43		0.85		0.83	0.88	2.12		1.89	2.35
**Family affluence**																	
More affluent (ref)	0.79		0.78	0.80	0.03		0.02	0.03		0.85		0.84	0.85	0.02		0.02	0.02
Less affluent	0.74		0.73	0.75	0.03		0.03	0.04		0.80		0.80	0.81	0.03		0.02	0.03
aRR	0.94		0.93	0.95	1.32		1.26	1.38		0.95		0.94	0.96	1.34		1.27	1.42
**Can talk to family about problems**																	
Strongly agree (ref)	0.77		0.76	0.78	0.03		0.03	0.03		0.83		0.82	0.84	0.02		0.02	0.02
Strongly disagree	0.79		0.77	0.80	0.03		0.02	0.03		0.83		0.82	0.85	0.02		0.02	0.02
aRR	1.02		1.00	1.04	0.91		0.82	1.00		1.01		0.99	1.02	0.95		0.86	1.04

Predicted values adjusted for age, type of school and location

aRR=adjusted risk ratio

^*^ Respondents who reported identifying as non-binary or two-spirited, described their gender differently, or preferred not to answer

^**^ The predicted proportions and aRR are based on ordered logistic regressions. To facilitate interpretation, the table only presents the proportions and aRR for the first outcome (e.g., never) and last outcome (e.g., often or almost always). The patterns for the other values were consistent with this interpretation

**Fig. 1:**
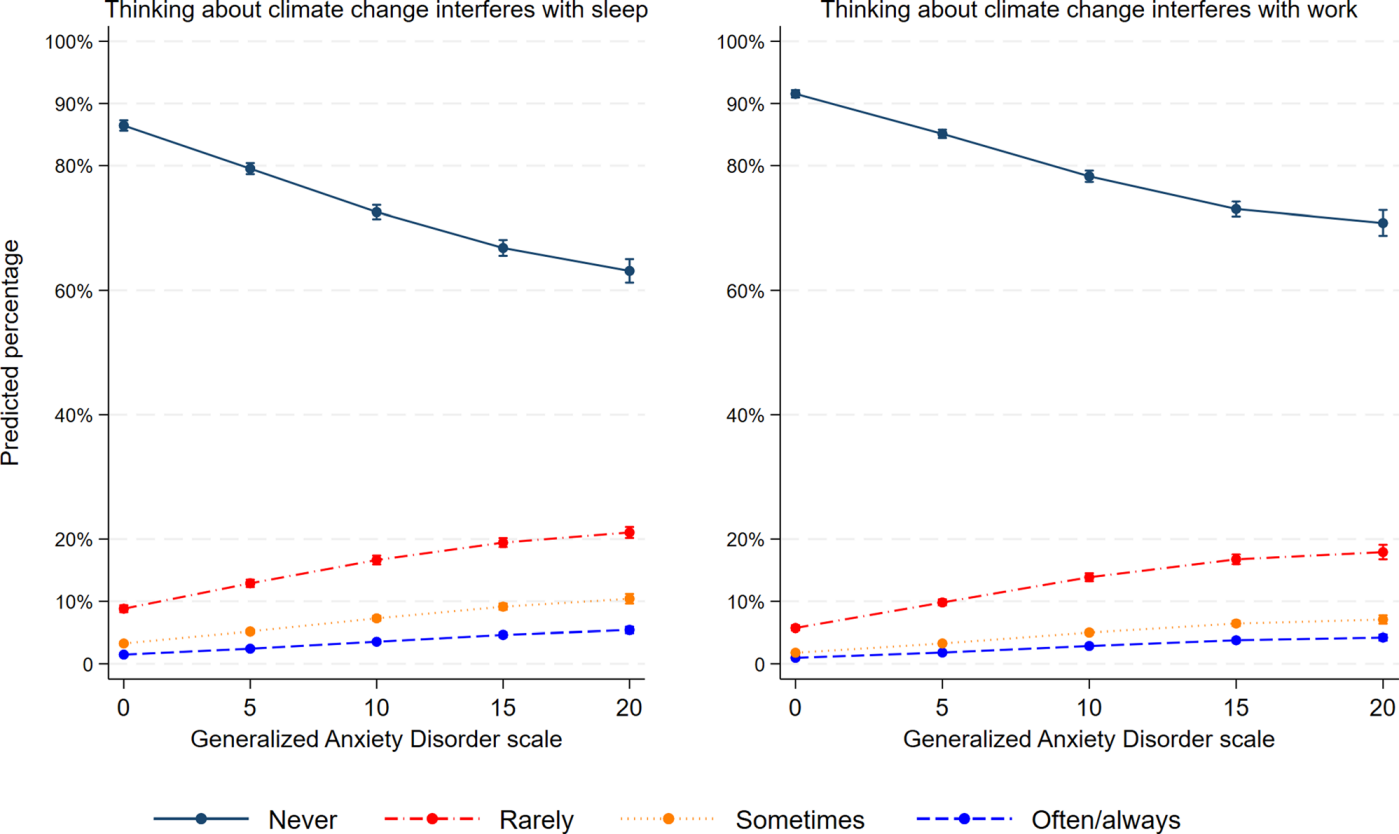
Association between anxiety and climate-related impairments amongst adolescents in Quebec (Canada). **Adjusted for age, gender, type of school and location

Overall, climate anxiety interfered less in the lives of adolescents who were less anxious and were better connected to their school. Figure 1 illustrates that for every one unit increase in anxiety the estimated proportion of adolescents who has sleep difficulty because they think of climate change gradually increased. For example, the predicted percentage of adolescents who often or almost always had difficulty sleeping was 2.4% [CI95: 2.3–2.6] for adolescents who scored 5 on the anxiety scale. The predicted value increased to 4.6% [CI95: 4.3- 5.0] for those who scored 15 on the anxiety scale and 5.4% [4.9–5.9] for those who scored 20. Figure 1 shows a similar pattern between anxiety symptoms and interference with the ability to do work due to climate change.

**Fig. 2:**
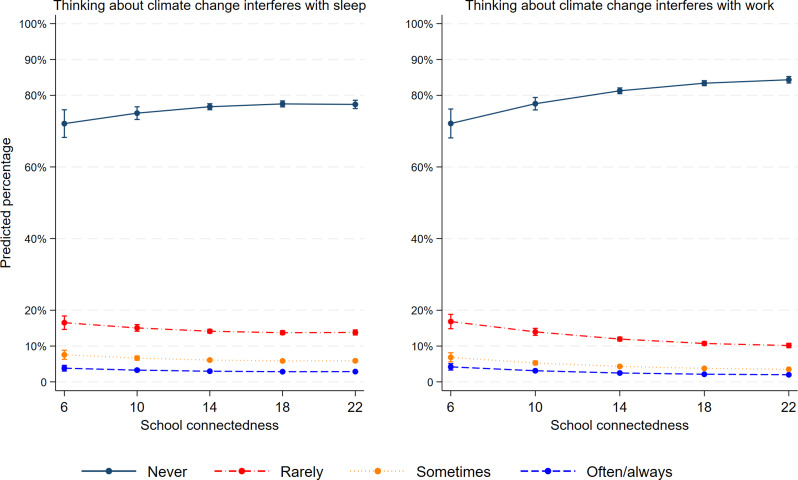
Association between school connectedness and climate-related impairments amongst adolescents in Quebec (Canada). **Adjusted for age, gender, type of school and location

School connectedness was negatively associated with both cognitive-emotional impairment and functional impairment related to climate anxiety. Figure 2 shows that for every one unit increase in the school connectedness score, the estimated proportion of adolescents who had sleep difficulty related to climate change slightly decreased. For example, the predicted percentage of adolescents who sometimes have difficulty sleeping was 7.6% [CI95: 6.3–8.8] for adolescents who have a score of 6 on the school connectedness scale and 5.9% [CI95: 5.5–6.3] for those who have a score of 22 on school connectedness. Figure 2 also illustrates that as the scores of school connectedness increased, the predicted percentages of adolescents who experience interference with the ability to do work slightly decreased.

### Environmental engagement

Self-efficacy and behavior change related to climate change were more common among girls (respectively RR = 1.23; CI95 [1.18, 1.27] and RR = 1.13; CI95 [1.08, 1.18]) and adolescents who identified as neither male nor female (RR = 1.41; CI95 [1.29; 1.52] and RR = 1.33; CI95 [1.22, 1.45]), compared to boys. Family affluence was not significantly associated with the belief that they could do something to address the problem or trying to reduce behaviors (RR = 1.02; CI95 [0.98; 1.05] and RR = 1.02; CI95 [0.99, 1.05]).

Table 3 shows that adolescents who strongly felt they could not talk to their family about their problems were 18% less likely to believe they could do something about climate change compared to those who strongly felt that they could talk with their families (RR = 0.82; CI95 [0.76, 0.88]). However, the ability to talk to their family about problems did not appear to affect the likelihood that they change their behaviors (RR = 0.94; CI95 [0.87, 1]).

**Table 3:** Predicted values and risk ratios for behavioral engagement amongst adolescents in Quebec (Canada)

	Believe they can do something									Try to change behaviors							
	Never				Often or almost always					Never				Often or almost always			
	Proportion*		95% CI		Proportion		95% CI			Proportion		95% CI		Proportion		95% CI	
**Gender**																	
Boys (ref)	0.48		0.47	0.50	0.13		0.12	0.14		0.45		0.44	0.47	0.19		0.18	0.20
Girls	0.43		0.41	0.45	0.16		0.15	0.17		0.42		0.40	0.44	0.21		0.20	0.22
aRR**	0.88		0.86	0.90	1.23		1.18	1.27		0.92		0.89	0.95	1.13		1.08	1.18
Neither boy nor girl^#^	0.38		0.36	0.41	0.18		0.17	0.20		0.36		0.34	0.39	0.25		0.23	0.27
aRR	0.79		0.75	0.85	1.41		1.29	1.52		0.80		0.75	0.86	1.33		1.22	1.45
**Family affluence**																	
More affluent (ref)	0.45		0.44	0.47	0.15		0.14	0.16		0.44		0.42	0.45	0.20		0.19	0.21
Less affluent	0.45		0.43	0.46	0.15		0.14	0.16		0.43		0.41	0.45	0.20		0.19	0.22
aRR	0.99		0.97	1.01	1.02		0.98	1.05		0.98		0.96	1.00	1.02		0.99	1.05
**Can talk to family about problems**																	
Strongly agree (ref)	0.45		0.43	0.46	0.15		0.14	0.16		0.44		0.4	0.46	0.19		0.18	0.2
Strongly disagree	0.50		0.48	0.53	0.12		0.11	0.14		0.46		0.4	0.49	0.18		0.17	0.2
aRR	1.13		1.08	1.17	0.82		0.76	0.88		1.04		0.99	1.09	0.94		0.87	1

Predicted values adjusted for age, type of school and location

**aRR = adjusted risk ratio

#Respondents who reported identifying as non-binary or two-spirited, described their gender differently, or preferred not to answer

** The predicted proportions and aRR are based on ordered logistic regressions. To facilitate interpretation, the table only presents the proportions and aRR for the first outcome (e.g., never) and last outcome (e.g., often or almost always). The patterns for the other values were consistent with this interpretation

**Fig. 3:**
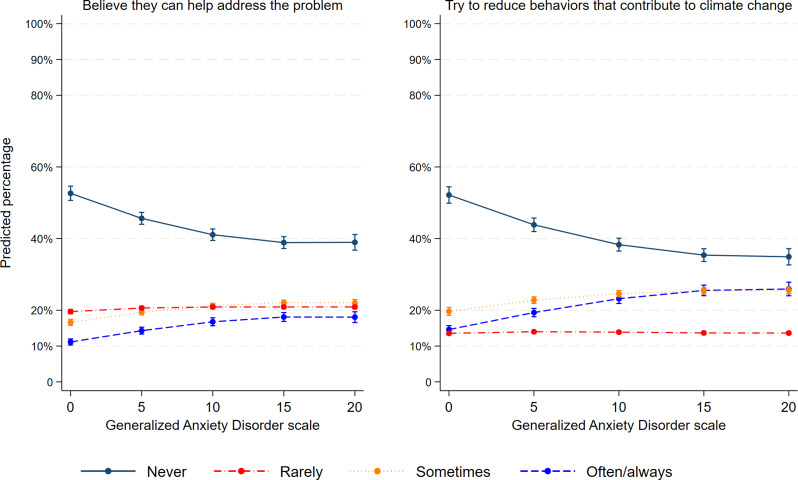
Association between symptoms of general anxiety and behavioral engagement amongst adolescents in Quebec (Canada). **Adjusted for age, gender, type of school and location

Figure 3 plots the positive relation between anxiety and the two measures of behavioral engagement (i.e., self-efficacy and pro-environmental engagement). Adolescents with more symptoms of general anxiety were more likely to believe they could do something. For example, 14.3% [CI95 13.3%, 15.3%] of adolescents with an anxiety score of 5 reported often or almost always believing that they can do something to address the problem. In contrast, 18% [CI95 16.6% 19.6%] of adolescents with an anxiety score of 20 often or almost always believed that they could do something. Similarly, for each one-unit increase in anxiety, adolescents were more likely to try to reduce their behaviors that contribute to climate change often or almost always (see Fig. 3).

**Fig. 4:**
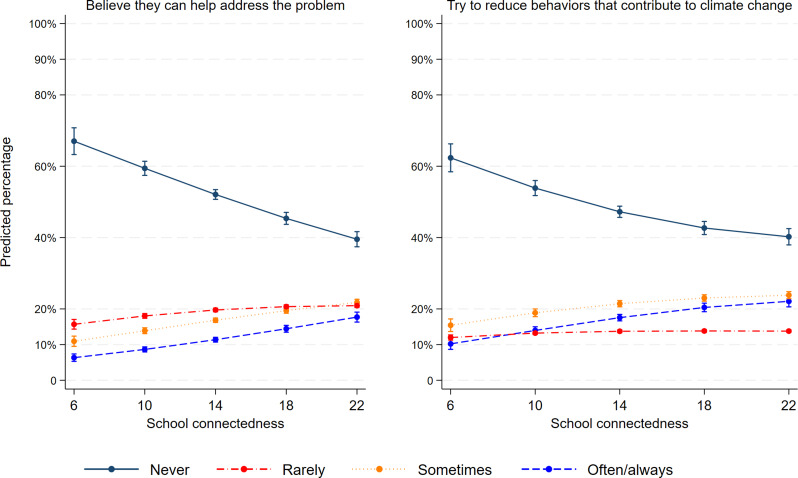
Association between school connectedness and behavioral engagement amongst adolescents in Quebec (Canada). ** Adjusted for age, sex, type of school and location

Figure 4 plots the relation between school connectedness and the two measures of behavioral engagement. For every one unit increase in school connectedness, the estimated proportion of adolescents who believed that they can help address the problem of climate change increased. For example, only 6.3% [CI95 5.3%, 7.4%] of adolescents with a score of school connectedness of 6 reported often or almost always believing that they can do something to address the problem. In contrast, 17.7% [CI95 16.3%, 19.1%] of adolescents with a school connectedness score of 22 often or almost always believed that they could do something. Similarly, attempts to change their behavior were more frequent in adolescents who had a stronger sense of connectedness to their school.

**Fig. 5:**
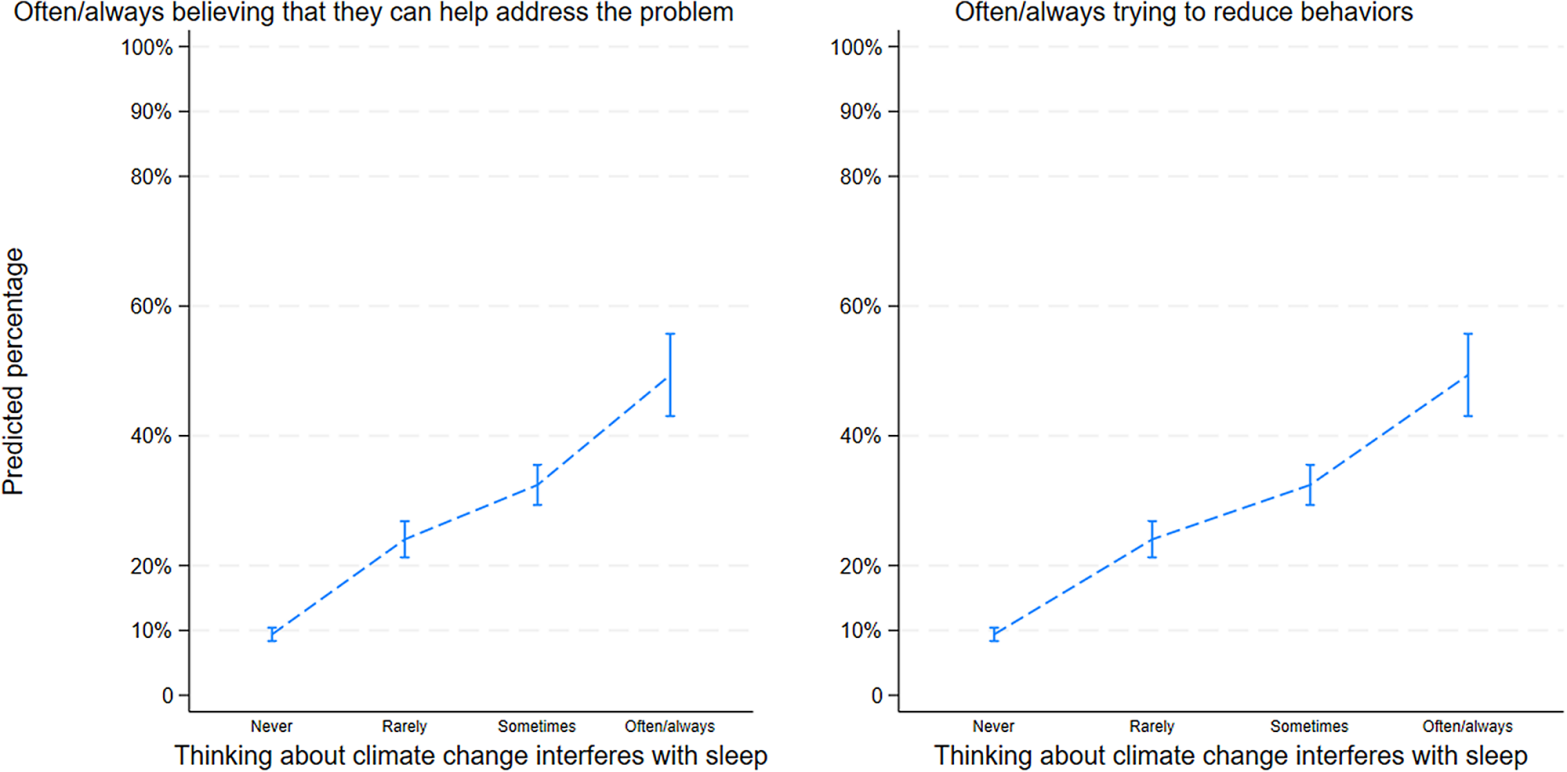
Association between climate-related impairment and behavioral engagement amongst adolescents in Quebec (Canada). **Adjusted for age, gender, type of school and location. *** To facilitate interpretation, the figure presents selected results from the ordered logistic regressions

Adolescents with more climate anxiety were more likely to believe that they could do something to help address the problem and to try to change their behaviors. Figure 5 shows that as the frequency of interference with sleep increased, the predicted percentage of adolescents who often or always believed that they can help address the problem increased. More specifically, amongst adolescents who never have sleep difficulty due to concerns about climate change, only 9.4% [CI95 8.4%, 10.4%] believed that they can help address the problem of climate change often or always. In contrast, the predicted percentage of those who often or always believed that they can help address climate change was 49% [CI95 43.0%, 55.7%] amongst those who often or always have sleep difficulty due to concerns about climate change. Similarly, Fig. 5 illustrates that for those who experienced interference with sleep more often, the predicted percentage of adolescents who often or always tried to reduce their behaviors was higher.

**Fig. 6:**
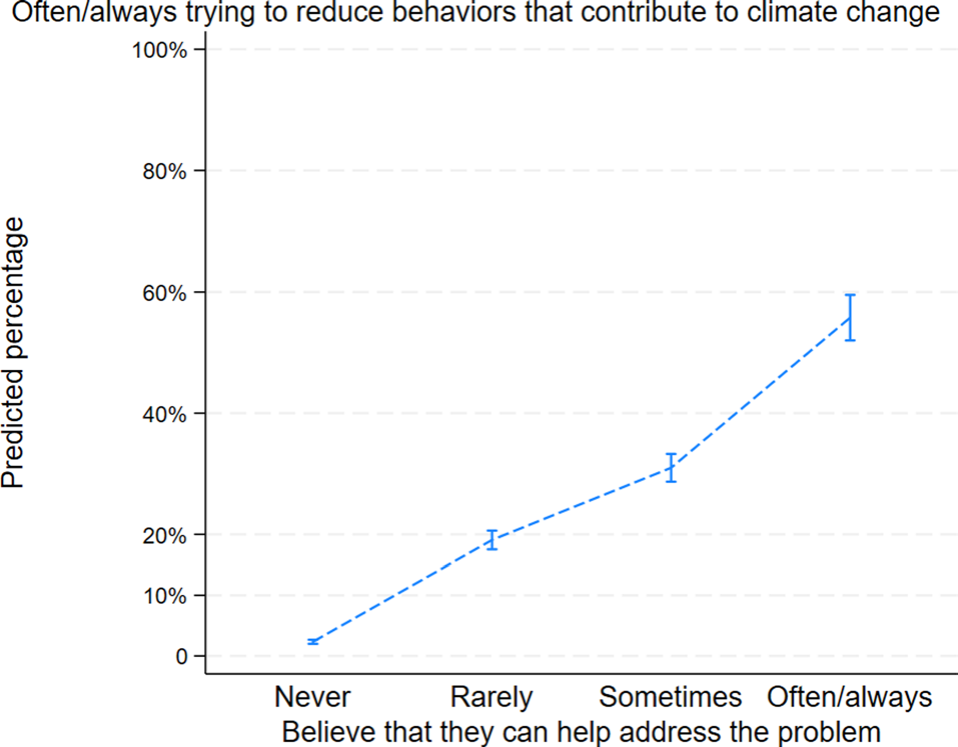
Relationship between the feeling of self-efficacy and pro-environmental behavior amongst adolescents in Quebec (Canada). **Adjusted for age, gender, type of school and location. ***This figure is based on the ordered logistic regressions that were conducted to analyze the relationship between self-efficacy and pro-environmental behavior. To facilitate interpretation, the figure only presents the curve for those who often/always try to reduce their behavior that contribute to climate change. The patterns for the other values of pro-environmental behavior were consistent with this interpretation

Figure 6 illustrates the role of self-efficacy in the adolescents’ attempts to reduce their behaviors that contributed to climate change. Adolescents who believed they could help address the problem of climate change were more likely to try to change their behavior often/always. For example, amongst adolescents who never believe that they can help address the problem, only 2.3% [CI95 2%, 2.7%] try to reduce their behaviors often or always. In contrast, amongst adolescents who often or always believe that they can help address the problem of climate change, 55.8% [CI95 52%, 60%] try to reduce their behaviors often or always.

## Discussion

This study presents the characteristics of Canadian adolescents who are experiencing impairments associated with climate change and who adopt pro-environment behaviors in a large sample of adolescents. The finding that 9% of adolescents experience difficulty sleeping related to climate anxiety and that 6% have interference with work at least sometimes highlights the importance of this public health issue and calls for greater attention from researchers, public health agencies, school specialists, and healthcare professionals.

Our findings support our hypothesis and are consistent with Clayton & Karazsia’s [5] study suggesting that females are more likely than males to display behavioral engagement. To date, few studies on climate anxiety have recognised the non-binary nature of gender; our study provides new insight showing that, compared to male adolescents, those who do not identify as either male or female are significantly more likely to experience impairments, to believe they can contribute, and to try to change their behavior. Future studies should try to replicate this new finding and explore environmental anxiety among cis-gender and gender minority populations longitudinally as adolescents transition through school. One potential explanation is that adolescents who question their gender are also more likely to question social norms and practices related to the environment. Alternatively, adolescents who report identifying as neither male nor female may also experience more mental health issues (e.g., depression) which has been shown to be associated with climate anxiety [8]. Other explanations regarding the gender differences may be related to level of connectedness to nature, or to media exposure.

The study also supports existing evidence on experiential inequalities regarding climate change [42]. We found that impairment due to climate anxiety was higher among adolescents from less affluent families. Some authors suggest that negative outcomes of climate change might be more salient for disadvantaged and vulnerable families [1]. It is also possible that disadvantaged adolescents who want to act may not have the agency or resources to do so, and this limited power may exacerbate climate anxiety.

The study helps to fulfill a knowledge gap, highlighted by Crandon et al. [1], regarding the psychological traits that are associated with climate anxiety and engagement. Adolescents who were more anxious were more likely to experience difficulty sleeping or interference with their ability to work. Those who felt connected to the school were less likely to experience climate anxiety. This is consistent with the literature highlighting the psychosocial characteristics that can influence climate anxiety. Costa et al. [43] found that children who were more insecurely attached to their parents (i.e., who had lower levels of trust and communication with their parents) had greater anxiety following exposure to Hurricane Katrina. Similarly, Crandon et al. [1] argued that not providing a safe space where adolescents can engage in dialogue about climate change isolates them within their own experience of anxiety. Adolescents should be encouraged to maintain peer connections, particularly during climate-change-related stressors [1].

The study’s findings support our hypothesis that adolescents who experience impairments are more likely to believe in their ability to help solve the problem and to try to change their behaviors that contribute to climate change. This finding is consistent with previous studies conducted on adult populations [6, 7, 13, 44]. Moreover, the study highlights the positive association between adolescents’ belief that they can do something to help address the problem and pro-environmental behaviors. Likewise, in the literature on emotions, hope has also been associated with pro-environmental behaviors. For example, Stevenson and Peterson [45] found that climate change concern and hope were positively related to pro-environmental behaviors while despair was negatively related to such behaviors. Similarly, Ojala [46] found that climate change hope predicted pro-environmental behavior among adolescents in Sweden. According to Rand [47], hope and self-efficacy have some conceptual overlap, as both are goal-directed and future-oriented. Together, these studies do not support the hypothesis that climate anxiety leads to eco-paralysis by inhibiting people from taking effective action [25].

The results of this study have important implications for practice and policy. First, the prevalence of cognitive-emotional and functional impairment due to climate anxiety amongst adolescents calls for more adaptation measures and action programs targeting this issue within the education system. School counselors could provide guidance and support to students who often have difficulty sleeping or doing schoolwork in order to better manage their climate anxiety and adopt coping strategies that enable them to remain functional. Teachers and counselors may have to be trained in environmental pedagogy to better address the environmental crisis that is increasingly publicized and affecting some adolescents. Also, the parents and families of adolescents experiencing high levels of impairment may need support to better manage and alleviate this anxiety.

The findings are useful to identify vulnerable groups that are more likely to experience impairments due to climate anxiety (e.g., females, individuals who identify as neither males nor females, those from less affluent families). As the threat of climate change increases, adolescents experiencing high levels of climate anxiety may continue to increase. It will be important to conduct longitudinal studies to assess whether the prevalence of climate anxiety intensifies over time and to understand the characteristics of students among whom it escalates faster than others.

The findings can also be useful to develop campaigns targeting groups that are less likely to try to reduce their climate-negative behaviors, such as adolescent males. It is noteworthy that 57% of the adolescents in this sample never or rarely tried to reduce their behaviors that contributed to climate change, while 66% never or rarely believed they could do something to help address the problem. Social media platforms could be useful to reach specific groups of adolescents and raise their awareness [48].

Our findings also shed light on some avenues that can be used to promote environmental engagement. Improving adolescents’ self-efficacy or beliefs in their capacity to help address the issue may be a key strategy to promote pro-environmental actions. Anderson et al. [34] argued that empowerment programs for adolescents could focus on psychosocial issues such as managing stress, obtaining family support, negotiating with peers, and dealing with uncomfortable emotions. Similarly, Wang et al. [49] advocated for purposefully structuring messages to increase human agency and facilitate climate action. It may also be useful to determine whether climate self-efficacy could promote environmental actions among other age groups that experience powerlessness [50]. In the years to come, public health researchers and professionals should explore and test school interventions that can address the pressing climate crisis, promote environmental pedagogy and empower adolescents to take much needed action in a constructive manner, without fueling climate anxiety.

### Limitations

The study has certain limitations related to its cross-sectional design. While we were able to identify factors correlated with climate anxiety, causal inference was not possible. Moreover, climate anxiety is a rapidly evolving phenomenon that is influenced not only by the collective awareness of climate change, but also by the health crisis. The study presented a snapshot of this phenomenon at a specific point in time, but the issue will continue to evolve among adolescents. Also, our analysis is based on the indicators available in the COMPASS 2022 survey. These provide an initial but incomplete overview of the attitudes of Canadian secondary school students towards climate change. Certain factors that could predispose young people to believe they can be agents of change could not be included. For example, future research could consider cultural differences, local norms, and parental beliefs and practices. Moreover, it should be noted that the measure of self-efficacy used a temporal scale (i.e., believing at least sometimes that they could do something against climate change). This approach differs from previous measures of self-efficacy [51]. In the standard methodology for measuring self-efficacy beliefs, responses range from (“Cannot do”) to (“Highly certain can do”) [51]. Future studies should explore different scales of self-efficacy related to climate change to provide a more comprehensive understanding of this phenomenon. The study was conducted in Quebec (Canada) and should also be replicated in other settings.

## Conclusion

Climate anxiety causes both cognitive-emotional impairment and functional impairment during adolescence, a vital period for laying the foundations of good health. This study revealed cognitive and experiential inequalities regarding climate anxiety. As climate change increasingly impacts our livelihoods in the coming years, it will be important to follow the evolution of climate anxiety among adolescents with different characteristics and living in different contexts. Future research should continue to explore the association between a young person’s relationship with their environment and their concerns, attitudes, and engagement in relation to climate change. Young people who feel more empowered to take action regarding climate change are also those who are trying to change their behaviors. Capacity-building training programs could be useful to educate adolescents about their power to act, without causing interference with their sleep or work, and to incite them to participate in the fight against climate change in a constructive way.

## Electronic supplementary material

Below is the link to the electronic supplementary material.

Supplementary Material 1

## Data Availability

The datasets used and/or analyzed during the current study are available from the corresponding author on reasonable request submitted via the following online application form: https://uwaterloo.ca/compass-system/information-researchers.

## Abbreviations

COMPASS: The Cannabis use, Obesity, Mental health, Physical activity, Alcohol use, Smoking, and Sedentary behavior Study
CI: Confidence intervals
RR: Risk ratios
