# Supplementary material for: Adolescents’ impairment due to climate anxiety is associated with self-efficacy and behavioral engagement: a cross-sectional analysis in Quebec (Canada)
Source: BMC Public Health. 2024 Oct 30;24:3009. doi: 10.1186/s12889-024-20333-y (PMC11526591; doi:10.1186/s12889-024-20333-y)
Supplement: Supplementary file 1 — Supplementary Material 1 [file 12889_2024_20333_MOESM1_ESM.docx]

**Supplementary file 1**

Figure S1. Flow chart of the samples


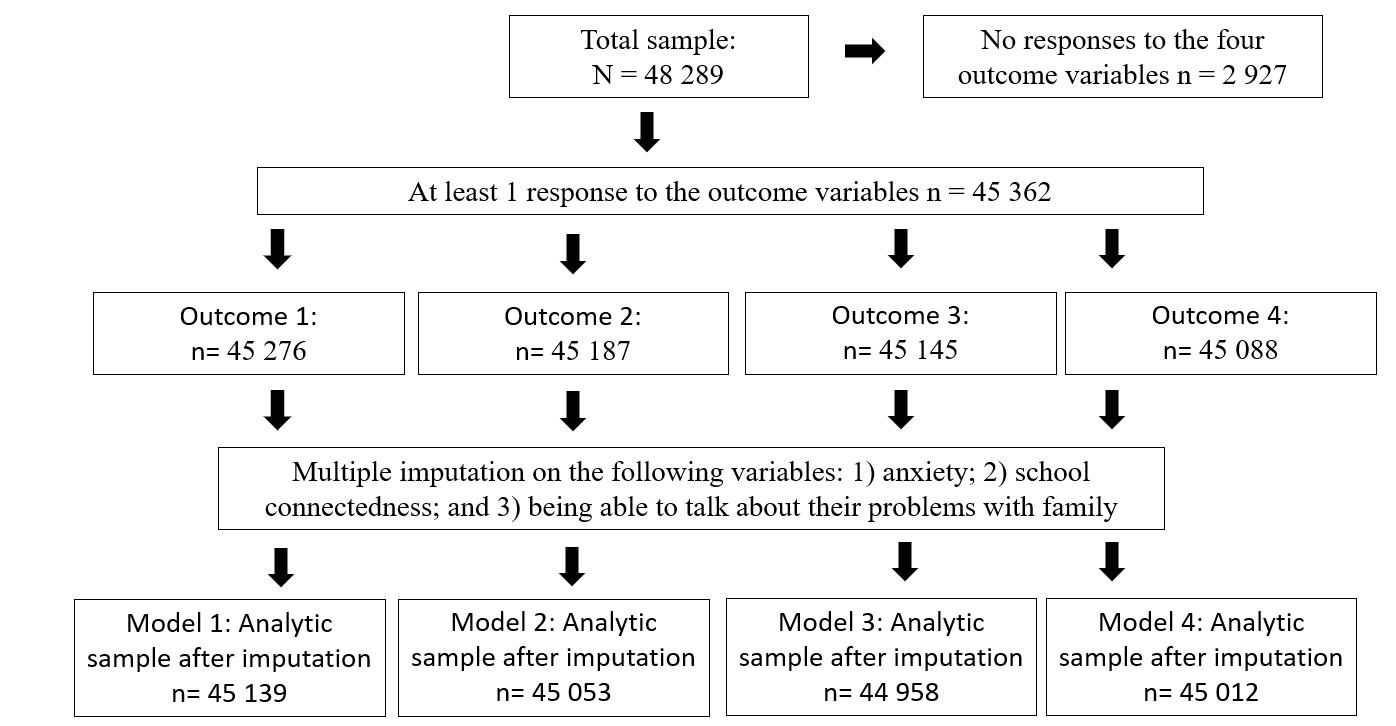


Table S2. Missing data

| **Variables** | N (%) | Missing |
| --- | --- | --- |
| Age (years) | 45 296 | 66 |
| Gender | 45 256 | 106 |
| Family affluence | 45 321 | 41 |
| School location | 45 362 | 0 |
| School type | 45 362 | 0 |
| School connectedness | 44 943 | 419 |
| Anxiety level | 43 655 | 1 707 |
| Can talk to family about problems | 45 010 | 352 |
| Difficulty sleeping | 45 276 | 86 |
| Interference with ability to work | 45 187 | 175 |
| Believe they can do something | 45 145 | 217 |
| Try to change behaviors | 45 088 | 274 |
|  |  |  |
